# Education as a moderator of middle-age cardiovascular risk factor—old-age cognition relationships: testing cognitive reserve hypothesis in epidemiological study

**DOI:** 10.1093/ageing/afab228

**Published:** 2022-02-02

**Authors:** Paula Iso-Markku, Jaakko Kaprio, Noora Lindgrén, Juha O Rinne, Eero Vuoksimaa

**Affiliations:** Institute for Molecular Medicine Finland (FIMM), Helsinki Institute of Life Science (HiLIFE), University of Helsinki, Helsinki, Finland; HUS Diagnostic Center, Clinical Physiology and Nuclear Medicine, University of Helsinki and Helsinki University Hospital, Helsinki, Finland; Institute for Molecular Medicine Finland (FIMM), Helsinki Institute of Life Science (HiLIFE), University of Helsinki, Helsinki, Finland; Clinicum, Department of Public Health, University of Helsinki, Helsinki, Finland; Turku PET Centre, University of Turku, Turku, Finland; Turku PET Centre, University of Turku and Turku University Hospital, Turku, Finland; Institute for Molecular Medicine Finland (FIMM), Helsinki Institute of Life Science (HiLIFE), University of Helsinki, Helsinki, Finland

**Keywords:** Cognition, cardiovascular risk, education, twins, resilience, older people

## Abstract

**Background:**

higher educational attainment and less midlife cardiovascular risk factors are related to better old-age cognition. Whether education moderates the association between cardiovascular risk factors and late-life cognition is not known. We studied if higher education provides resilience against the deteriorative effects of higher middle-age body mass index (BMI) and a combination of midlife cardiovascular risk factors on old-age cognition.

**Methods:**

the study population is the older Finnish Twin Cohort (*n* = 4,051, mean age [standard deviation, SD] = 45.5 years [6.5]). Cardiovascular risk factors and education were studied at baseline with questionnaires in 1975, 1981 and/or 1990 (participation rates of 89, 84 and 77%, respectively). Cognition was evaluated with telephone interviews (participation rate 67%, mean age [SD] =73.4 [2.9] years, mean follow-up [SD] = 27.8 [6.0] years) in 1999–2017. We studied the main and interactive effects of education and BMI/dementia risk score on late-life cognition with linear regression analysis. The study design was formulated before the pre-defined analyses.

**Results:**

years of education moderated the association between BMI with old-age cognition (among less educated persons, BMI-cognition association was stronger [*B* = −0.24 points per BMI unit, 95% CI −0.31, −0.18] than among more educated persons [*B* = −0.06 points per BMI unit, 95% CI −0.16, 0.03], *P*_interaction_ < 0.01). There was a similar moderating effect of education on dementia risk score consisting of cardiovascular risk factors (*P* < 0.001).

**Conclusions:**

our results support the cognitive reserve hypothesis. Those with higher education may tolerate the deteriorative effects of midlife cardiovascular risk factors on old-age cognition better than those with lower education.

## Key Points

- Education moderates the relationship between midlife cardiovascular risk factors and old-age cognition.
- Negative association between cardiovascular risk factors and old-age cognition is stronger in those with lower education.
- Higher educated persons tolerate better the deteriorative effects of midlife cardiovascular risk factors on old-age cognition.

## Introduction

Middle-age cardiovascular (CV) risk factors [1] including body mass index (BMI; [2, 3]) are robustly associated with poorer old-age cognition and increased risk of dementia. Higher education is related to better old-age cognition and is a consistent protective factor against dementia [4]. Indeed, education can be considered as a lifetime exposure increasing cognitive reserve (CR) helping to withstand risk factors of dementia [5]. CR is a property of the brain that allows to withstand age-related or disease-related brain changes. CR hypothesis is supported if the negative association between risk factors of dementia and old-age cognition is attenuated in those with higher education compared to those with lower education [5]. For example, three population-based studies indicated that education halved the risk of dementia attributed to carrying the Apolipoprotein E gene ε4-allele [6].

Only few studies have investigated if education moderates the association between CV risk factors and dementia or late-life cognition. Two cross-sectional studies showed that education or general cognitive ability were associated with attenuated obesity-related cognitive dysfunction [7, 8]. Another cross-sectional study gave some evidence that education moderated the negative association between white matter hyperintensities and episodic memory such that individuals with higher level of education showed weaker negative association [9]. In a large prospective register study from Denmark, neither education nor young adult general cognitive ability moderated the association between BMI and dementia in men [10]. However, lower BMI at 19 years was associated with higher subsequent dementia risk during the 44-year follow-up [10].

The long preclinical period of Alzheimer’s disease—the most common cause of dementia—predisposes short follow-up studies to reverse causality. On the other hand, risk factors may not have yet developed if measured at a very early age. When studying diseases with long preclinical periods, risk factors should be studied before the disease process has started. The association between a risk factor and the disease may and does often alternate across life course [3]. Hence, the moderation effect of education on the relationship between midlife CV risk factors and old-age cognition remains elusive, despite the evidence that education does not moderate the association of young adult BMI and dementia [10].

In this cohort study, we tested if education moderates the associations of midlife BMI and dementia risk score including multiple CV risk factors with late-life cognition. Our hypothesis was that education moderates these associations such that the associations are stronger in those with lower levels of education compared those with higher levels of education. In addition, we conducted quasi-experimental design to study if within twin-pair differences in education are related to within twin-pair differences in late-life cognition in twin pairs with the same level of BMI or dementia risk score: compared to studies in unrelated individuals, these within twin-pair comparisons provide more control for unmeasured shared environmental and genetic effects.

## Methods

### Participants

**Figure 1:**
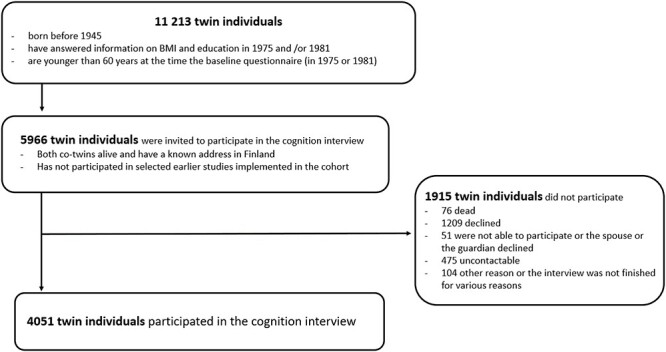
Flowcart of the participants of the older Finnish Twin Cohort included in this study.

Participants were from the Finnish Twin Cohort (FTC) comprising of all the same-sex twin pairs born in Finland before 1958 with both co-twins alive in 1967 [11]. Participants filled out questionnaires on health and health-related behaviours in 1975, 1981 (all cohorts) and 1990 (cohorts born in 1930 or later). The response rates were high: 89, 84 and 77%, respectively [11]. From 1999 onwards, twins aged at least 65 years were invited to participate in a telephone cognition interview (participation rate 67%). Twins born before 1938 were interviewed in 1999–2007 and twins born in 1938–1944 were interviewed in 2013–2017 [12]. The number of twins born in 1916–1944 with baseline information on educational level and BMI was 14,922. A total of 11,213 were younger than 60 years at baseline and of them, 4,051 individuals had telephone interview data (Figure 1). Zygosity was confirmed with deoxyribonucleic acid (DNA) analysis for 60% of the analysis sample while the remaining zygosities were confirmed with a validated questionnaire showing over 90% accuracy [13].

### Risk and protective factors

Education, height, weight and CV risk factors were self-reported in 1975, 1981 and/or 1990. We used a mean BMI from 1975 and 1981 or from one time point in case of missing data. The dementia risk score based mainly on CV risk factors we used was a modified version of Cardiovascular Risk Factors, Aging and Incidence of Dementia (CAIDE) dementia risk score [14, 15]. CAIDE score is a dementia risk score combining educational level, age, sex and a multiple of CV risk factors: hypercholesterolaemia, hypertension, obesity and low physical activity [14]. CAIDE dementia risk score in FTC has been described previously in detail [15] and we used this score without education in this study (referred to as ‘CAIDE without education’, ‘CAIDE-modified’ or ‘CAIDE-m’).

### Late-life cognition

Two telephone screening instruments for cognitive impairment were used in this study to evaluate cognition: telephone interview for cognitive status (TICS; [16]) and telephone assessment of cognition (TELE; [17]). They both are highly valid in the Finnish population and discriminate well between normal cognition and dementia [18]. They test, for example, for orientation, serial subtraction, verbal memory immediate recall and delayed recall, object naming and attention. We used total cognitive score, a combination of these two tools notwithstanding the overlapping items, in this study [19] as a measure of cognitive functioning. Higher score denotes better cognition (range 0–51).

### Statistical methods

We compared the differences in baseline characteristics in participants and non-participants of cognition interview with a *t*-test for continuous variables and with χ^2^-test for categorical values (both adjusted for clustered twin data). We used linear regression analysis without (main effects) and with (moderation effects) interaction terms. A robust cluster estimator was used to correct for the relatedness within twin pairs [20]. All the analyses were adjusted for age and sex. In interaction models, we centred age, years of education and BMI. We present the results in beta estimates and 95% confidence intervals (CIs). The pre-specified outcome variable, total cognitive score, had a near normal distribution by visual inspection.

Education was used both as a continuous (years of education) and a categorical variable (0–11 years of education or ≥12 years of education ergo primary and secondary versus tertiary education). In addition, we provide the results with a standardised education measure in supplementary analyses (according to the International Standard Classification of Education [ISCED] responding 0–6 years of education, 7–10 years, 11–12 years, 13–15 years and ≥ 15 years; for further details see [21]).

In the analyses with BMI, we excluded the twins aged 60 or more at the time of the relevant questionnaire (in 1981 primary or in 1975 if the answer from 1981 was missing) to minimise possibility of reverse causation. For the analyses with CAIDE-m, we excluded the twins aged at least 60 years in 1981 or 1990 depending on which year they reported information on cholesterol [15].

In within-family analyses, we selected twin pairs concordant for high CV risk and performed within-twin pair analyses in which the differences in educational level were regressed on within-twin pair differences in cognition [22]. In these analyses, BMI was dichotomous with the World Health Organization threshold for overweight (25) used as a cut-off. For CAIDE-m, the cut-off was set at 5, which was the integer nearest to the mean in the cohort. We performed similar within-family analyses for twin pairs concordant for low level of education (<12 years) and regressed within-twin pair differences in CV risk on within-twin pair differences in cognition. As sensitivity analyses, we also performed similar analyses for twin pairs concordant for low CV risk or high education and discordant for education or CV risk level, respectively. Further, by using interaction analysis, we formally tested if these associations are similar in twin pairs concordant for high versus low risk factors. We used Stata 16.0 in all analyses (StataCorp LLC) and we followed STrengthening the Reporting of OBservational studies in Epidemiology (STROBE) guidelines for cohort studies.

## Results

### Demographics

At baseline, mean age (standard deviation [SD], range) was 45.5 [6.5, 31.0–59.9] years (47.1% women) and mean [range] BMI was 24.5 [16.5–41.3] kg/m^2^ and participants had on average [SD] 7.9 [2.9] years of education (see Table 1 and Supplementary Data, Supplementary Table S1 and Drop-out analysis are available at *Age and Ageing* online). At the end of the follow-up (mean [SD] = 27.8 [6.0] years, range 16.5–41.7), at a mean age [SD] of 73.4 [2.9] the mean [SD] total cognitive score was 40.1 [5.3] (range 5–51).

**Table 1:** Baseline characteristics of the study cohort (*n* = 4,051)

	**The older finnish twin cohort**
**Age** (years, mean, SD)	45.5 (6.5)
**Gender**	
Men (*n*, %)	2141 (52.8)
Women (*n*, %)	1910 (47.2)
**BMI** (kg/m2, mean, SD)	24.5 (3.0)
**Years of education (mean, SD)**	7.9 (2.9)
**ISCED categories of years of schooling (** *n* **, %)**	
1: 0–6 years	1761 (43.5)
2: 7–10 years	1836 (45.3)
3: 11–12 years	10 (0.3)
4: 13–15 years	142 (3.5)
5: ≥15 years	302 (7.5)
**CAIDE without education (0–12)** (mean, SD) ^a^	4.8 (2.3)
**Twin pairs concordant for education** (at the accuracy of 1 year)	696
0–11 years of education	614 (88.2)
≥ 12 years of education	82 (11.8)
**Twin pairs concordant for BMI** (at the accuracy of the same weight category [<25, ≥25])	888
BMI < 25 BMI ≥ 25	612 (68.9) 276 (31.1)
**Twin pairs concordant for CAIDE-m** (at the accuracy of the same dichotomous category (<5, ≥5))	403
CAIDE-m < 5 CAIDE-m ≥ 5	171 (42.4) 232 (57.6)

*Abbreviations:* SD, standard deviation; BMI, body mass index.

^a^Number of twin individuals with CAIDE score without education = 2,359.

**Figure 2:**
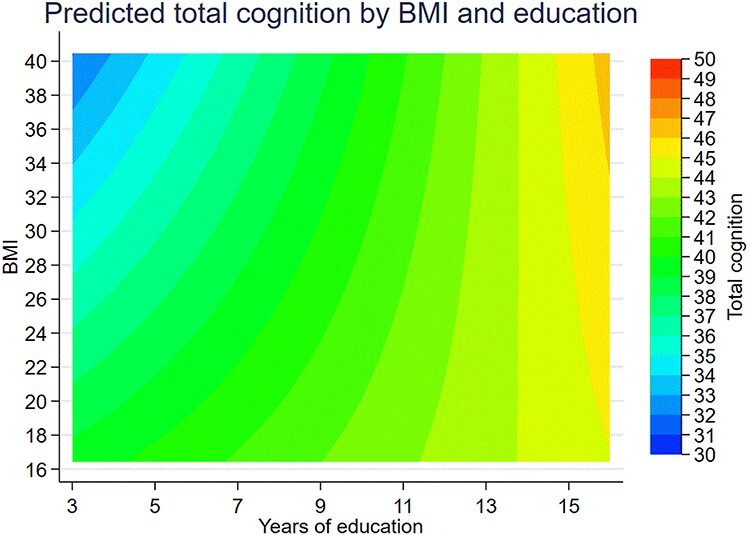
Contour plot of total cognitive score by BMI and continuous education. *Abbreviation:* BMI, body mass index. ^a^  *n* = 4,051. Unadjusted BMI and years of education are used in this figure.

**Figure 3:**
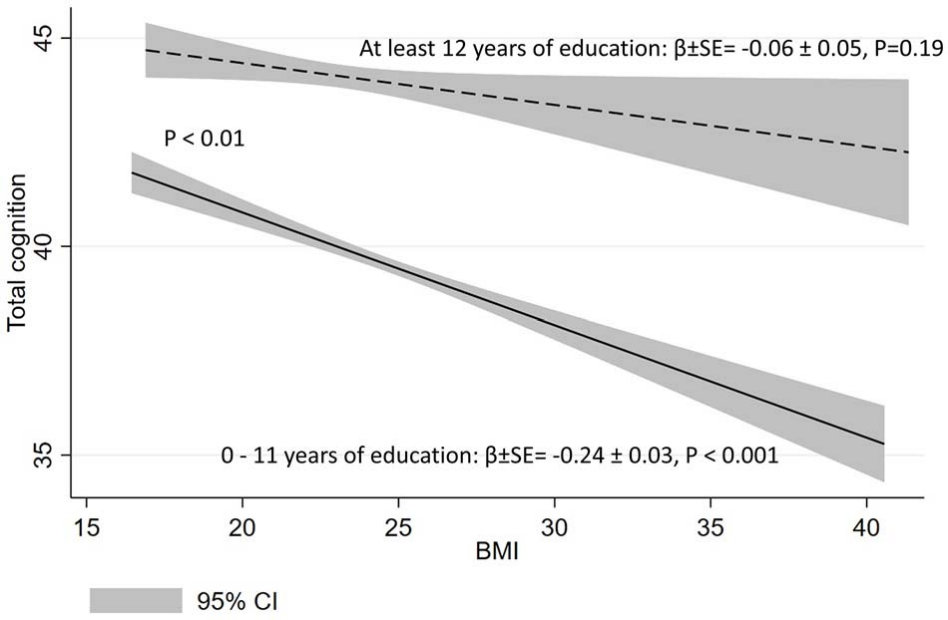
Total cognitive score by BMI and categorical education. *Abbreviations:* BMI, body mass index; β, beta coefficient; SE, standard error; CI, confidence intervals.

### Main effects

Years of education in midlife was positively associated with total cognitive score in late life when adjusted for age and sex (*B* = 0.63, 95% CI: 0.59, 0.68). Middle-age BMI was negatively associated with late-life cognition when adjusted for age and sex (*B* = −0.18, 95% CI −0.23, −0.12).

Midlife CAIDE-m was negatively associated with late-life cognition when adjusted for age and sex: *B* = −0.48, 95% CI: −0.56, −0.39 (*n* = 2,363) and also when adjusted for age, sex and years of education: *B* = −0.37, 95% CI: −0.45, −0.29 (*n* = 2,359).

### Interaction analyses

Education moderated the effect of BMI on late-life cognition: the effect of BMI was decreased as a function of higher educational level both with education as a continuous variable (*P*_interaction_ < 0.001, Figure 2) and with education as a dichotomous variable (*P*_interaction_ < 0.01, Figure 3) or five-category ISCED variable (see Supplementary Data, Appendix, Supplementary Table S2, available at *Age and Ageing* online).

Further, education also moderated the effect of CAIDE-m on old-age cognition (*P*_interaction_ < 0.001, see Supplementary Data, Supplementary Figure S1, Appendix, available at *Age and Ageing* online). CAIDE-m had stronger association with cognition in lower-educated individuals compared to those with higher education.

### Within-twin pair analyses

With regard to BMI, the within-twin pair differences in education were related to within-twin pair differences in late-life cognition in twin pairs where both co-twins were overweight or obese (Table 2). Similarly, in sensitivity analyses of twin pairs where both co-twins were normal or underweight, within twin-pair differences in education were related to within twin-pair differences in cognition but the effect size for the association was smaller than in obese or overweight twin pairs (Table 2). In twin pairs concordant for high CAIDE-m, within twin-pair differences in education were related to within twin-pair differences in cognition. In sensitivity analyses of twin pairs concordant for low CAIDE-m (0–5), within-twin pair differences in education were not significantly related to within—twin pair differences in late-life cognition (Table 2). However, interaction analyses did not show significant interaction for different association in twin pairs concordant for high versus low CV risk factors (*P*_interaction for BMI and education_ = 0.28 and *P*_interaction for CAIDE-m and education_ = 0.14). In twin pairs concordant for the length of education, the within-twin pair differences in BMI or CAIDE-m were not associated with late-life cognition (Table 2).

**Table 2:** The within-differences in education and cardiovascular risk predicting cognition^a^

**Predictor**	**Risk factor for concordance (*N*’s)**	**Twin pairs concordant for high dementia risk**	**Twin pairs concordant for low dementia risk**
**Education**	**BMI (<25 versus ≥ 25)** (BMI < 25: *n* = 1225 twin individuals, 612 full twin pairs; BMI ≥ 25: *n* = 553 twin individuals, 276 full twin pairs)	0.71 (0.29 to 1.14)	0.46 (0.24 to 0.68)^*^
	**CAIDE-m (<5 versus 5–12)** (CAIDE-m < 5: *n* = 342 twin individuals, 171 full twin pairs; CAIDE-m 5–12: *n* = 464 twin individuals, 232 full twin pairs)	0.66 (0.26 to 1.07)	0.29 (−0.01 to 0.58)
**BMI**	**Education (<12 versus ≥ 12)** (Education <12: *n* = 1230 twin individuals, 614 full twin pairs; Education ≥12: *n* = 165 twin individuals, 82 full twin pairs)	−0.13 (−0.36 to 0.10)	−0.33 (−0.86 to 0.20)
**CAIDE-m**	**Education (<12 versus ≥ 12)** (Education <12: *n* = 766 twin individuals, 276 full twin pairs; Education ≥12: *n* = 114 twin individuals, 43 full twin pairs)	−0.19 (−0.63 to 0.25)	0.15 (−1.18 to 1.48)

*Abbreviations:* BMI, body mass index; CAIDE-m, modified version of Cardiovascular Risk Factors, Aging and Dementia risk score.

^a^All analyses are adjusted for age. Age is centered. Concordant for education signifies that the members of a twin pair have the same years of education. Concordant for BMI signifies that the members of a twin pair have the same weight category (<25, ≥25). Concordant for CAIDE-score without education signifies that the members of a twin pair have the same CAIDE score without education category (scores 0–5 and scores 5–12). Interaction analyses did not show significant interaction for different association in twin pairs concordant for high versus low CV risk factors (*P*_interaction_ for BMI and education = 0.28 and *P*_interaction_ for CAIDE-m and education = 0.14).

## Discussion

Education, midlife BMI and self-reported CAIDE-m were independent predictors of old-age cognition in our population-based sample with an average follow-up of 28 years. We found that education moderated the associations between midlife BMI and CAIDE-m with late-life cognition. Negative association between midlife BMI and cognition became smaller as a function of higher educational attainment and BMI was no longer significantly associated with cognition in individuals with at least 12 years of education. Education moderated similarly the association between midlife CAIDE-m, a measure including multiple midlife CV risk factors, and late-life cognition suggesting that dementia risk evaluation based on CV risk factors may work differently in those with less and more education.

In epidemiological research, CR hypothesis is supported if education moderates the association between risk factors and cognition [5]. The between-family results of this study support the CR hypothesis by showing that those with higher education appear to tolerate the deteriorative effects of midlife CV risk factors on old-age cognition better than those with lower education. This finding is in accordance with neural differences in mild Alzheimer’s disease in low versus high-educated individuals at the same level of cognitive performance: those with 15 years of education had increased amyloid and lower glucose uptake compared to those with 6 years of education [23].

In within-family analyses of twin pairs concordant for being overweight, twins with higher education had better old-age cognition compared to their co-twins with lower education. The beneficial effect of education on cognition was stronger among twin pairs concordant for being overweight than in twin pairs concordant for being normal of underweight. However, interaction analysis showed no difference in the strength of the association in high BMI versus normal BMI concordant pairs suggesting that education is positively associated with old-age cognition in general when controlling for middle-age BMI as well as unmeasured shared genetic and environmental influences.

In twin pairs concordant for low education (as well as for high education), the within-twin pair differences in BMI or CAIDE-m were not associated with within–twin pair differences in late-life cognition suggesting that the association between midlife CV risk factors and old-age cognition is confounded by shared genetic and environmental effects. Also, an earlier study has suggested confounding by genetic and environmental factors in the association of BMI and dementia [24]. This means that the association between middle-age risk factors and old-age cognition does not arise from individual lifestyle choices made in midlife but from genetics and early life experiences affecting those lifestyle choices.

Within-family results provide more evidence for the causal effect of educational attainment on old-age cognition whereas the support for causality independent of genetic and environmental effects was not observed for middle-age CV risk factors—cognition associations. Our results are in line with studies in dementia discordant twin pairs indicating that education rather than BMI or other CV risk factors is related to risk of dementia or cognitive decline when controlling for genetic and environmental effects [15, 26], although contradicting findings exist [25, 27, 28]. Similarly, other studies have provided more causal evidence for the educational attainment—dementia association [29, 30] with some evidence indicating the importance of higher general cognitive ability as the mechanism behind the education-dementia association [31, 32].

Our results imply that in a natural situation without interventions, CV risk factors do not convey any additional risk for worse late-life cognitive performance in individuals with high level of education over and above the environment and genetics shared with old-age cognition. We emphasise, however, that the importance of CV risk factors should not be overlooked. Our study results emphasise the importance of primary and secondary education in dementia prevention [33]. Recent evidence synthesis from Lancet Commission attributed 7% of dementia prevalence to low education and only 1% of dementia prevalence to overweight and obesity [33] Further, it was estimated that 40% of world-wide dementias could be prevented or postponed by affecting 12 modifiable factors (low education, hypertension, hearing impairment, smoking, obesity, depression, physical inactivity, diabetes, and low social contact, excessive alcohol consumption, traumatic brain injury and air pollution; [33]). Potential for dementia prevention is suggested to be higher in low- and middle-income countries [29]. At the same time, the incidence of dementia is stabilising or decreasing in high-income countries [34], which may be explained by the decreasing prevalence of these 12 modifiable risk factors with education level in front.

Our results support a complex pattern of protective and risk factors associations with dementia. According to our results, CV risk factors could explain even a larger proportion of dementias in low-educated individuals. On the other hand, in countries with high level of education, the proportion of dementias, which can be prevented or postponed, may be significantly smaller. It must be also noted that both dementia and lifestyle factors are considerably heritable [35]. Our results highlighted how closely bound the midlife CV risk factors are with environment and genetics shared with cognition, illustrating the difficulty in changing long-term lifestyle habits. Interventions to change CV factors may need to be well targeted and intensive [36].

The cut-off for high level of education in this study was 12 years. This particular cut-off is well in line with earlier research showing that the age at which this level of education is achieved (19 years in Finland) is likely to be also the threshold at which education-associated gains in general cognitive ability are likely to plateau [32]. Our result contrasting those with less than 12 years of education and those with 12 years or more education supports the suggestion that for better dementia prevention, everyone should have access to primary and secondary education [29]. Our data were limited in individuals with high level of education and even more scarce in persons with a university degree. Future studies should systematically look if very high level of education grants even more reserve against deteriorating effects of vascular risk on cognition.

The strengths of our study included the midlife baseline data and long follow-up of 28 years minimising the possibility of reverse causation that occurs when CV risk factors are measured in preclinical period of dementia [37]. The results from this large population-based sample with high participation rates are well generalisable in the Finnish population because twins have been shown not to differ from the general population in terms of several traits including behaviour [38] or morbidity and mortality [39]. The prevalence of dementia in this study is also comparable to the prevalence of dementia in the Finnish general population [15]. Twin data allowed also to investigate if the protective effect of education is evident in high CV risk individuals who are matched according to shared environmental and genetic background; quasi-experimental setting that cannot be used in studies on unrelated individuals.

CV factors were self-reported, but Finns have been shown to be reliable self-reporters of these measures [40, 41]. TELE/TICS cannot replace in-person neurological or neuropsychological examinations and may be affected by hearing problems. Drop-out analysis indicated selection: the participants of the telephone screening study were more educated. This phenomenon is common in research, and the difference in this study was small. Further, our sample was not selected in terms of midlife BMI. The effect sizes could be stronger in an unselected study cohort.

Our study supported the CR hypothesis. Higher education may allow for better tolerance of CV risk factors with regard to old-age cognition. In twin pairs concordant for high CV risk, the within-twin pair differences in education were associated with within-twin pair differences in cognition highlighting the importance of education in dementia prevention.

## Supplementary Material

aa-21-0715-File002_afab228

## Data Availability

Data are available on reasonable request. Because of the consent given by study participants and the high degree of identifiability, data cannot be made publicly available. Data are available through the Institute for Molecular Medicine Finland (FIMM) Data Access Committee (DAC) for authorised researchers who have IRB/ethics approval and an institutionally approved study plan. For more details, please contact the FIMM DAC (fimm-dac@helsinki.fi).

## References

[1] Lourida I, Hannon E, Littlejohns TJ et al. Association of lifestyle and genetic risk with incidence of dementia. JAMA 2019; 322: 430–7. 31302669 10.1001/jama.2019.9879 PMC6628594

[2] Lee CM, Woodward M, Batty GD et al. Association of anthropometry and weight change with risk of dementia and its major subtypes: a meta-analysis consisting 2.8 million adults with 57 294 cases of dementia. Obes Rev 2020; 21: e12989. 10.1111/obr.12989. PMC7079047 31898862

[3] Kivimäki M, Luukkonen R, Batty GD et al. Body mass index and risk of dementia: analysis of individual-level data from 1.3 million individuals. Alzheimers Dement 2018; 14: 601–9. 10.1016/j.jalz.2017.09.016 PMC5948099 29169013

[4] Meng X, D'Arcy C. Education and dementia in the context of the cognitive reserve hypothesis: a systematic review with meta-analyses and qualitative analyses. PLoS One 2012; 7: e38268. 10.1371/journal.pone.0038268. 22675535 PMC3366926

[5] Stern Y, Arenaza-Urquijo EM, Bartrés-Faz D et al. Whitepaper: defining and investigating cognitive reserve, brain reserve, and brain maintenance. Alzheimers Dement 2020; 16: 1305–11. 30222945 10.1016/j.jalz.2018.07.219 PMC6417987

[6] Wang HX, Gustafson DR, Kivipelto M et al. Education halves the risk of dementia due to apolipoprotein ε4 allele: a collaborative study from the Swedish brain power initiative. Neurobiol Aging 2012; 33: e1–7. 22056199 10.1016/j.neurobiolaging.2011.10.003

[7] Kirton JW, Dotson VM. The interactive effects of age, education, and BMI on cognitive functioning. Neuropsychol Dev Cogn B Aging Neuropsychol Cogn 2016; 23: 253–62. 26667889 10.1080/13825585.2015.1082531 PMC4683610

[8] Galioto RM, A losco ML, Spitznagel MB et al. Cognitive reserve preserves cognitive function in obese individuals. Neuropsychol Dev Cogn B Aging Neuropsychol Cogn 2013; 20: 684–99. 23339557 10.1080/13825585.2012.762972

[9] Zahodne LB, Mayeda ER, Hohman TJ et al. The role of education in a vascular pathway to episodic memory: brain maintenance or cognitive reserve? Neurobiol Aging 2019; 84: 109–18. 31539647 10.1016/j.neurobiolaging.2019.08.009 PMC6960324

[10] Osler M, Okholm GT, Sørensen TIA et al. Body mass index in young adulthood and risk of subsequent dementia at different levels of intelligence and education in Danish men. Eur J Epidemiol 2020; 35: 843–50. 32728913 10.1007/s10654-020-00665-w

[11] Kaprio J, Bollepalli S, Buchwald J et al. The older Finnish twin cohort - 45 years of follow-up. Twin Res Hum Genet 2019; 22: 240–54. 31462340 10.1017/thg.2019.54

[12] Lindgren N, Kaprio J, Rinne JO et al. Immediate verbal recall and familial dementia risk: population-based study of over 4000 twins. J Neurol Neurosurg Psychiatry 2019; 90: 90–7. 30315123 10.1136/jnnp-2018-319122

[13] Sarna S, Kaprio J, Sistonen P et al. Diagnosis of twin zygosity by mailed questionnaire. Hum Hered 1978; 28: 241–54. 566252 10.1159/000152964

[14] Kivipelto M, Ngandu T, Laatikainen T et al. Risk score for the prediction of dementia risk in 20 years among middle aged people: a longitudinal, population-based study. Lancet Neurol 2006; 5: 735–41. 16914401 10.1016/S1474-4422(06)70537-3

[15] Iso-Markku P, Kaprio J, Lindgrén N, Rinne J, Vuoksimaa E. Middle-age dementia risk scores and old-age cognition: a quasi-experimental population-based twin study with over 20-year follow-up. J Neurol Neurosurg Psychiatry 2020; 92: 323–30. 33154181 10.1136/jnnp-2020-324009 PMC7892379

[16] Brandt JSM, Folstein M. The telephone interview for cognitive status. Neuropsychiatry Neuropsychol Behav Neurol 1998; 1: 111–7.

[17] Gatz M, Reynolds CA, John R et al. Telephone screening to identify potential dementia cases in a population-based sample of older adults. Int Psychogeriatr 2002; 14: 273–89. 12475088 10.1017/s1041610202008475

[18] Järvenpää T, Rinne JO, Räihä I et al. Characteristics of two telephone screens for cognitive impairment. Dement Geriatr Cogn Disord 2002; 13: 149–55. 11893836 10.1159/000048646

[19] Vuoksimaa E, Rinne JO, Lindgren N et al. Middle age self-report risk score predicts cognitive functioning and dementia in 20-40 years. Alzheimers Dement (Amst) 2016; 4: 118–25. 27752535 10.1016/j.dadm.2016.08.003 PMC5061466

[20] Williams RL . A note on robust variance estimation for cluster-correlated data. Biometrics 2000; 56: 645–6. 10877330 10.1111/j.0006-341x.2000.00645.x

[21] Okbay A, Beauchamp JP, Fontana MA et al. Genome-wide association study identifies 74 loci associated with educational attainment. Nature 2016; 533: 539–42. 27225129 10.1038/nature17671 PMC4883595

[22] McGue M, Osler M, Christensen K. Causal inference and observational research: the utility of twins. Perspect Psychol Sci 2010; 5: 546–56. 21593989 10.1177/1745691610383511 PMC3094752

[23] Kemppainen NM, Aalto S, Karrasch M et al. Cognitive reserve hypothesis: Pittsburgh compound B and fluorodeoxyglucose positron emission tomography in relation to education in mild Alzheimer's disease. Ann Neurol 2008; 63: 112–8. 18023012 10.1002/ana.21212

[24] Xu WL, Atti AR, Gatz M et al. Midlife overweight and obesity increase late-life dementia risk: a population-based twin study. Neurology 2011; 76: 1568–74. 21536637 10.1212/WNL.0b013e3182190d09 PMC3100125

[25] Gatz M, Mortimer JA, Fratiglioni L et al. Accounting for the relationship between low education and dementia: a twin study. Physiol Behav 2007; 92: 232–7. 17597169 10.1016/j.physbeh.2007.05.042 PMC2225456

[26] Karlsson IK, Lehto K, Gatz M et al. Age-dependent effects of body mass index across the adult life span on the risk of dementia: a cohort study with a genetic approach. BMC Med 2020; 18: 131. 10.1186/s12916-020-01600-2. 32513281 PMC7282125

[27] Xiong GL, Plassman BL, Helms MJ et al. Vascular risk factors and cognitive decline among elderly male twins. Neurology 2006; 67: 1586–91. 17101888 10.1212/01.wnl.0000242730.44003.1d

[28] Virta JJ, Heikkila K, Perola M et al. Midlife cardiovascular risk factors and late cognitive impairment. Eur J Epidemiol 2013; 28: 405–16. 23532744 10.1007/s10654-013-9794-y

[29] Larsson SC, Traylor M, Malik R et al. Modifiable pathways in Alzheimer's disease: Mendelian randomisation analysis. BMJ 2017; 359: j5375. 10.1136/bmj.j5375. 29212772 PMC5717765

[30] Wang Z, Meng L, Shen L et al. Impact of modifiable risk factors on Alzheimer's disease: a two-sample Mendelian randomization study. Neurobiol Aging 2020; 91: 167.e11–9. 10.1016/j.neurobiolaging.2020.02.018 32204957

[31] Anderson EL, Howe LD, Wade KH et al. Education, intelligence and Alzheimer's disease: evidence from a multivariable two-sample Mendelian randomization study. Int J Epidemiol 2020; 49: 1163–72. 32003800 10.1093/ije/dyz280 PMC7660137

[32] Kremen WS, Beck A, Elman JA et al. Influence of young adult cognitive ability and additional education on later-life cognition. Proc Natl Acad Sci U S A 2019; 116: 2021–6. 30670647 10.1073/pnas.1811537116 PMC6369818

[33] Livingston G, Huntley J, Sommerlad A et al. Dementia prevention, intervention, and care: 2020 report of the lancet commission. Lancet 2020; 396: 413–46. 32738937 10.1016/S0140-6736(20)30367-6 PMC7392084

[34] Roehr S, Pabst A, Luck T et al. Is dementia incidence declining in high-income countries? A systematic review and meta-analysis. Clin Epidemiol 2018; 10: 1233–47. 30271219 10.2147/CLEP.S163649 PMC6149863

[35] Polderman TJ, Benyamin B, de Leeuw CA et al. Meta-analysis of the heritability of human traits based on fifty years of twin studies. Nat Genet 2015; 47: 702–9. 25985137 10.1038/ng.3285

[36] Ngandu T, Lehtisalo J, Solomon A et al. A 2 year multidomain intervention of diet, exercise, cognitive training, and vascular risk monitoring versus control to prevent cognitive decline in at-risk elderly people (FINGER): a randomised controlled trial. Lancet 2015; 385: 2255–63. 25771249 10.1016/S0140-6736(15)60461-5

[37] Jack CR Jr, Knopman DS, Jagust WJ et al. Hypothetical model of dynamic biomarkers of the Alzheimer's pathological cascade. Lancet Neurol 2010; 9: 119–28. 20083042 10.1016/S1474-4422(09)70299-6 PMC2819840

[38] Pulkkinen L, Vaalamo I, Hietala R et al. Peer reports of adaptive behavior in twins and singletons: is twinship a risk or an advantage? Twin Res 2003; 6: 106–18. 12723997 10.1375/136905203321536236

[39] Kaprio J . The finnish twin cohort study: an update. Twin Res Hum Genet 2013; 16: 157–62. 23298696 10.1017/thg.2012.142 PMC4493754

[40] Haapanen N, Miilunpalo S, Pasanen M et al. Agreement between questionnaire data and medical records of chronic diseases in middle-aged and elderly Finnish men and women. Am J Epidemiol 1997; 145: 762–9. 9126003 10.1093/aje/145.8.762

[41] Tuomela J, Kaprio J, Sipilä PN et al. Accuracy of self-reported anthropometric measures - findings from the Finnish twin study. Obes Res Clin Pract 2019; 13: 522–8. 31761633 10.1016/j.orcp.2019.10.006 PMC9234778

