# Supplementary material for: Education as a moderator of middle-age cardiovascular risk factor—old-age cognition relationships: testing cognitive reserve hypothesis in epidemiological study
Source: Age Ageing. 2022 Feb 2;51(2):afab228. doi: 10.1093/ageing/afab228 (PMC8824709; doi:10.1093/ageing/afab228)
Supplement: aa-21-0715-File002_afab228 [file aa-21-0715-file002_afab228.docx]

**Education as a moderator of middle-age cardiovascular risk factor – old-age cognition relationships: testing cognitive reserve hypothesis in epidemiological study**

Supplementary Data

- Supplementary Table S1. The drop-out analyses
- Drop out analyses
- Supplementary Table S2. Regression coefficients and 95% confidence intervals of total cognitive score on body mass index by strata of International Standard Classification of Education (ISCED) educational categories
- Supplementary Figure S1. Contour plot of total cognitive score by CAIDE-m and continuous education

**Supplementary Table S1. The drop-out analyses**

|  | **Participants in the telephone cognition interview**  (n=4051) | **Non-participants in the telephone cognition interview**  (n=1364) | **P value for the difference between the groups^b^** |
| --- | --- | --- | --- |
| **Age** (years, mean, SD) | 45.5 (6.5) | 43.8 (6.9) | 0.000 |
| **Gender** |  |  |  |
| Men (n, %) | 2141 (52.8) | 599 (43.9) |  |
| Women (n, %) | 1910 (47.2) | 765 (56.1) | 0.000 |
| **BMI** (kg/m2, mean, SD) | 24.5 (3.0) | 24.3 (3.2) | 0.086 |
| **Years of education (mean, SD)** | 7.9 (2.9) | 7.1 (2.4) | 0.000 |
| **ISCED categories of years of schooling (n, %)** |  |  |  |
| 1: 0-6y | 1761 (43.5) | 779 (57.1) |  |
| 2: 7-10y | 1836 (45.3) | 503 (36.9) |  |
| 3: 11-12y | 10 (0.3) | 6 (0.4) |  |
| 4: 13-15y | 142 (3.5) | 17 (1.2) |  |
| 5: ≥15y | 302 (7.5) | 59 (4.3) | 0.000 |
| **CAIDE without education (0 – 12)**  (mean, SD) ^a^ | 4.8 (2.3) | 4.2 (2.3) | 0.000 |

Abbreviations: SD= standard deviation; BMI, body mass index; ISCED, International Standard Classification of Education; y, year; CAIDE, Cardiovascular Risk Factors, Aging and Incidence of Dementia risk score.

^a^ Number of twin individuals with CAIDE score without education = 2359 in participants and 559 in non-participants.

^b^ Statistical difference tested with t-test for continuous values (clustered for twin structure) and with χ2 test for categorical values (clustered for twin structure).

**Drop out analyses**

Baseline BMI did not differ significantly between the twins who participated in the cognition interview and the twins who did not participate in the cognition interview (means were 24.5 (SD=3.0) and 24.3 (SD=3.2), respectively). The twins who participated in the cognition interview were more educated (7.9 years of education, SD=2.9) than the twins who did not participate in the cognition interview (7.1 years of education, SD=2.4) and their modified Cardiovascular Risk Factors, Aging and Incidence of Dementia score (CAIDE-m) was higher.

**Supplementary Table S2. Regression coefficients and 95% confidence intervals of total cognitive score on body mass index by strata of International Standard Classification of Education (ISCED) educational categories^a^**

| **ISCED categories** | **n** | **β coefficients** | **Confidence intervals** |
| --- | --- | --- | --- |
| **1** | 1762 | -0.19 | -0.28, -0.09 |
| **2** | 1838 | -0.21 | -0.29, -0.13 |
| **3** | 10 | 0.14 | -0.79, 1.08 |
| **4** | 142 | -0.04 | -0.19, 0.10 |
| **5** | 303 | -0.07 | -0.19, 0.06 |

Abbreviations: BMI, body mass index; ISCED, International Standard Classification of Education.

n= 4051

^a^ Models are adjusted for age and sex and clustered for twin data. BMI and age are centered. Only individuals whose BMI and education were measured when they were less than 60 years old were included.

**Supplementary Figure S1. Contour plot of total cognitive score by CAIDE-m and continuous education^a^**


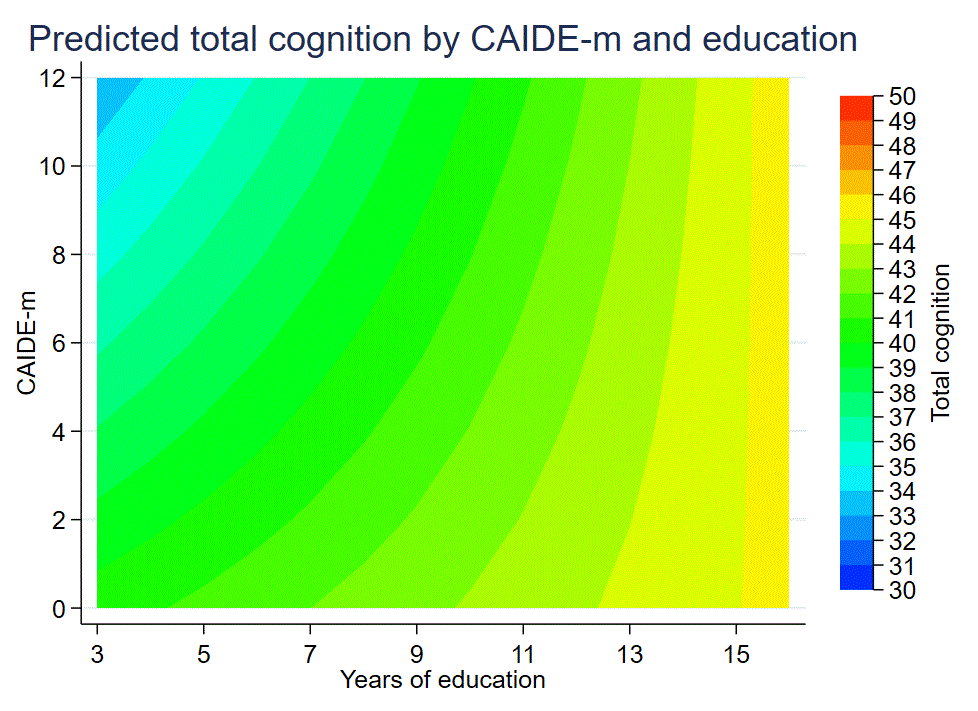


Abbreviations: CAIDE-m = Cardiovascular Risk Factors, Aging and Incidence of Dementia (CAIDE) score without education

^a^ n=4051. Unadjusted CAIDE-m and years of education are used in this figure.
